# Crystal structure of 2-[12-methyl-14-phenyl-10,13,14,16-tetra­aza­tetra­cyclo[7.7.0.0^2,7^.0^11,15^]hexa­deca-1(16),2,4,6,9,11(15),12-heptaen-8-yl­idene]propandi­nitrile

**DOI:** 10.1107/S1600536814024167

**Published:** 2014-11-08

**Authors:** Joel T. Mague, Shaaban K. Mohamed, Mehmet Akkurt, Hussein M. S. El-Kashef, Mustafa R. Albayati

**Affiliations:** a Department of Chemistry, Tulane University, New Orleans, LA 70118, USA; b Chemistry and Environmental Division, Manchester Metropolitan University, Manchester M1 5GD, England; c Chemistry Department, Faculty of Science, Minia University, 61519 El-Minia, Egypt; d Department of Physics, Faculty of Sciences, Erciyes University, 38039 Kayseri, Turkey; e Department of Chemistry, Faculty of Science, Assiut University, 71515 Assiut, Egypt; f Kirkuk University, College of Science, Department of Chemistry, Kirkuk, Iraq

**Keywords:** crystal structure, hepta­ene, propandi­nitrile, pyrazine scaffold compound, fused tetracyclic core

## Abstract

In the title mol­ecule, C_22_H_12_N_6_, the fused tetracyclic core shows a small lengthwise twist as indicated by the dihedral of 2.7 (2)° between the outer rings. In the crystal, mol­ecules stack along the *b*-axis direction *via* offset π-stacking [centroid–centroid distances = 3.5282 (13) and 3.5597 (14) Å] with the stacks weakly associated through C—H⋯N hydrogen bonds. The phenyl ring is rotationally disordered over two orientations with an occupancy ratio of 0.516 (4):0.484 (4).

## Related literature   

For the biological properties of pyrazine scaffold compounds, see: Kaliszan *et al.* (1985 ▶); Makino *et al.* (1990 ▶); Emary & Ibrahim (2006 ▶); Silva *et al.* (2010 ▶); Rusinov *et al.* (2005 ▶); Johnston & Kau (1993 ▶); Myadaraboina *et al.* (2010 ▶); Metobo *et al.* (2006 ▶). For use of pyrazines in industrial chemistry see: Rangnekar & Dhamnaskar, 1990 ▶). For the preparation of the title compound, see: El-Emary & El-Kashef (2013 ▶)
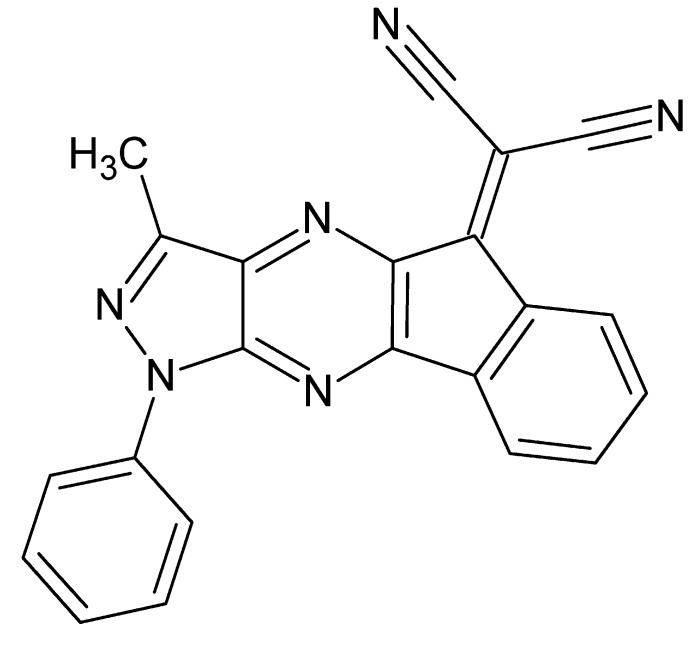


## Experimental   

### Crystal data  

C_22_H_12_N_6_
*M* *_r_* = 360.38
Monoclinic, 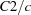

*a* = 35.968 (5) Å
*b* = 4.6483 (6) Å
*c* = 26.596 (3) Å
β = 129.6130 (12)°
*V* = 3425.5 (8) Å^3^
*Z* = 8
Mo *K*α radiation
μ = 0.09 mm^−1^
*T* = 150 K
0.21 × 0.13 × 0.07 mm

### Data collection  

Bruker SMART APEX CCD diffractometer
Absorption correction: multi-scan (*SADABS*; Bruker, 2014 ▶) *T*_min_ = 0.77, *T*_max_ = 0.99
15751 measured reflections
3921 independent reflections
2489 reflections with *I* > 2σ(*I*)
*R*_int_ = 0.050

### Refinement  

*R*[*F* ^2^ > 2σ(*F* ^2^)] = 0.053
*wR*(*F* ^2^) = 0.136
*S* = 1.03
3921 reflections
249 parameters
1 restraint
H-atom parameters constrained
Δρ_max_ = 0.31 e Å^−3^
Δρ_min_ = −0.20 e Å^−3^

### 

Data collection: *APEX2* (Bruker, 2014 ▶); cell refinement: *SAINT* (Bruker, 2014 ▶); data reduction: *SAINT*; program(s) used to solve structure: *SHELXT* (Sheldrick, 2008 ▶); program(s) used to refine structure: *SHELXL2014* (Sheldrick, 2008 ▶); molecular graphics: *DIAMOND* (Brandenburg & Putz, 2012 ▶); software used to prepare material for publication: *SHELXTL* (Sheldrick, 2008 ▶).

## Supplementary Material

Crystal structure: contains datablock(s) global, I. DOI: 10.1107/S1600536814024167/su5012sup1.cif

Structure factors: contains datablock(s) I. DOI: 10.1107/S1600536814024167/su5012Isup2.hkl

Supporting information file. DOI: 10.1107/S1600536814024167/su5012Isup3.cml

**Fig. 1.**
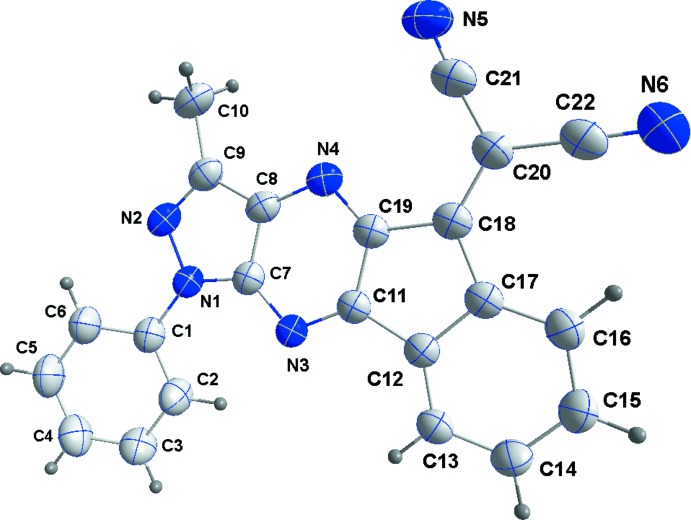
The molecular structure of the title molecule, showing the atom labelling. Displacement ellipsoids are drawn at the 50% probability level.

. DOI: 10.1107/S1600536814024167/su5012fig1.tif
The mol­ecular structure of the title mol­ecule, showing the atom labelling. Displacement ellipsoids are drawn at the 50% probability level.

**Fig. 2.**
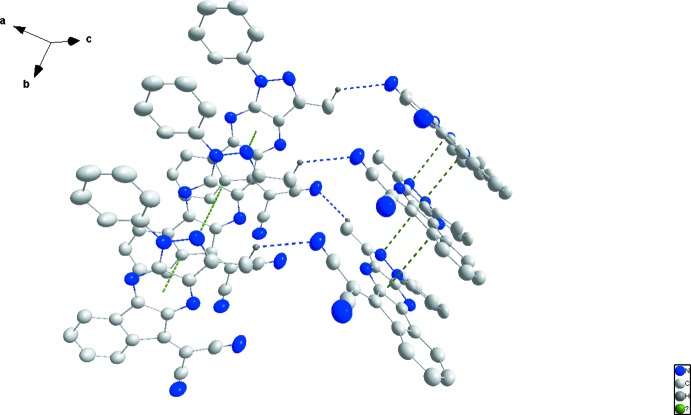
Portions of two neighboring stacks showing the offset π-stacking and C—H···N interactions (Table 1) as green and blue dotted line, respectively.

**Fig. 3.**
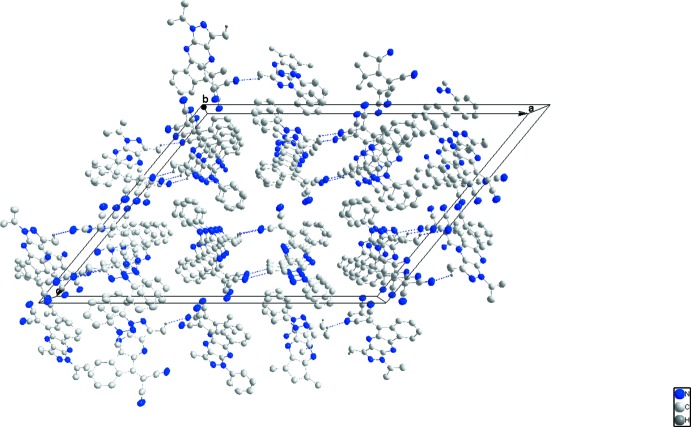
Crystal packing viewed along the b axis showing stacks of molecules connected by the weak C—H···N interactions (blue dotted lines; see Table 1 for details).

. DOI: 10.1107/S1600536814024167/su5012fig2.tif
Portions of two neighboring stacks showing the offset π-stacking and C—H⋯N inter­actions (Table 1) as green and blue dotted line, respectively.

b . DOI: 10.1107/S1600536814024167/su5012fig3.tif
Crystal packing viewed along the *b* axis showing stacks of mol­ecules connected by the weak C—H⋯N inter­actions (blue dotted lines; see Table 1 for details).

CCDC reference: 1032263

Additional supporting information:  crystallographic information; 3D view; checkCIF report

## Figures and Tables

**Table 1:** Hydrogen-bond geometry (, )

*D*H*A*	*D*H	H*A*	*D* *A*	*D*H*A*
C10H10*A*N5^i^	0.98	2.69	3.362(3)	126

Symmetry code: (i) 
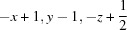
.

## supplementary crystallographic information

### S1. Comment

Over recent years there has been an increasing interest in the chemistry of pyrazine scaffold compounds due to their biological significance. Pyrazine ring is found in numerous pharmaceutically active compounds such as analgesic (Kaliszan *et al.*, 1985), antiallergic (Makino *et al.*, 1990), antibacterial (Emary & Ibrahim 2006), anti-inflammatory (Silva *et al.*, 2010), antiviral (Rusinov *et al.*, 2005), diuretic (Johnston & Kau, 1993), anticancer (Myadaraboina *et al.*, 2010), and anti-HIV (Metobo *et al*., 2006) medications. Other pyrazine derivatives are also used as fluorescent dyes or dispersed dyes for polyester fibers (Rangnekar & Dhamnaskar, 1990). As part of our investigations of pyrazine derivatives to compare their chemical and biological activities, we have undertaken the X-ray crystal structure analysis of the title compound.

In the title compound, Fig. 1, the fused 4-ring core of the title molecule is nearly planar with only a slight lengthwise twist as indicated by the dihedral angle between the N1/N2/C7/C8/C9 and C12–C17 rings of 2.7 (2)°.

In the crystal, molecules pack in columns along [010] which involve offset π-stacking in which atom N2 is 3.36 (4) Å from the centroid of the N3/C11/C19/N4/C8/C7 ring one unit cell translation in *b* above it while C17 is 3.41 (4) Å from the centroid of the N3/C11/C19/N4/C8/C7 ring one unit cell translation in *b* below it (Fig. 2). Adjacent stacks are weakly associated *via* C—H···N hydrogen bonds (Fig. 3 and Table 1) and are inclined at *ca* 43.5° in opposite directions from (010).

### S2. Experimental

The title compound was prepared according to the reported procedure (El-Emary & El-Kashef, 2013). Orange crystals suitable for X-ray diffraction were obtained by recrystallization of the reaction product from dimethylformamide (m.p. 587–589 K).

### S3. Refinement

C-bound H atoms were placed in calculated positions and treated as riding atoms, with C—H = 0.95–0.98 Å and with U_iso_(H) = 1.5U_eq_(C) for methyl H atoms and = 1.2U_eq_(C) for other H atoms. The phenyl ring attached to N1 is rotationally disordered over two sites with an occupancy ratio of 0.516 (4):0.484 (4). The components of the disorder were refined as rigid hexagons.

### Crystal data

**Table:**

C_22_H_12_N_6_	*F*(000) = 1488
*M**_r_* = 360.38	*D*_x_ = 1.398 Mg m^−^^3^
Monoclinic, *C*2/*c*	Mo *K*α radiation, λ = 0.71073 Å
*a* = 35.968 (5) Å	Cell parameters from 4540 reflections
*b* = 4.6483 (6) Å	θ = 2.3–27.4°
*c* = 26.596 (3) Å	µ = 0.09 mm^−^^1^
β = 129.6130 (12)°	*T* = 150 K
*V* = 3425.5 (8) Å^3^	Column, orange
*Z* = 8	0.21 × 0.13 × 0.07 mm

### Data collection

**Table:**

Bruker SMART APEX CCD diffractometer	3921 independent reflections
Radiation source: fine-focus sealed tube	2489 reflections with *I* > 2σ(*I*)
Graphite monochromator	*R*_int_ = 0.050
Detector resolution: 8.3660 pixels mm^-1^	θ_max_ = 27.5°, θ_min_ = 2.0°
φ and ω scans	*h* = −46→46
Absorption correction: multi-scan (*SADABS*; Bruker, 2014)	*k* = −6→6
*T*_min_ = 0.77, *T*_max_ = 0.99	*l* = −34→34
15751 measured reflections	

### Refinement

**Table:**

Refinement on *F*^2^	Primary atom site location: structure-invariant direct methods
Least-squares matrix: full	Secondary atom site location: difference Fourier map
*R*[*F*^2^ > 2σ(*F*^2^)] = 0.053	Hydrogen site location: inferred from neighbouring sites
*wR*(*F*^2^) = 0.136	H-atom parameters constrained
*S* = 1.03	*w* = 1/[σ^2^(*F*_o_^2^) + (0.0537*P*)^2^ + 1.9123*P*] where *P* = (*F*_o_^2^ + 2*F*_c_^2^)/3
3921 reflections	(Δ/σ)_max_ = 0.001
249 parameters	Δρ_max_ = 0.31 e Å^−^^3^
1 restraint	Δρ_min_ = −0.20 e Å^−^^3^

### Special details

**Table:**

Experimental. The diffraction data were collected in three sets of 400 frames (0.5° width in ω) at φ = 0, 120 and 240°. A scan time of 90 sec/frame was used.
Geometry. All e.s.d.'s (except the e.s.d. in the dihedral angle between two l.s. planes) are estimated using the full covariance matrix. The cell e.s.d.'s are taken into account individually in the estimation of e.s.d.'s in distances, angles and torsion angles; correlations between e.s.d.'s in cell parameters are only used when they are defined by crystal symmetry. An approximate (isotropic) treatment of cell e.s.d.'s is used for estimating e.s.d.'s involving l.s. planes.
Refinement. Refinement of *F*^2^ against ALL reflections. The weighted *R*-factor *wR* and goodness of fit *S* are based on *F*^2^, conventional *R*-factors *R* are based on *F*, with *F* set to zero for negative *F*^2^. The threshold expression of *F*^2^ > σ(*F*^2^) is used only for calculating *R*-factors(gt) *etc*. and is not relevant to the choice of reflections for refinement. *R*-factors based on *F*^2^ are statistically about twice as large as those based on *F*, and *R*- factors based on ALL data will be even larger. H-atoms attached to carbon were placed in calculated positions (C—H = 0.95 - 0.98 Å). All were included as riding contributions with isotropic displacement parameters 1.2 - 1.5 times those of the attached atoms. The phenyl ring attached to N1 is rotationally disordered over two sites in approximately equal amounts. The components of the disorder were refined as rigid hexagons.

### Fractional atomic coordinates and isotropic or equivalent isotropic displacement parameters (Å^2^)

**Table:**

	*x*	*y*	*z*	*U*_iso_*/*U*_eq_	Occ. (<1)
N1	0.69392 (6)	0.2255 (4)	0.36211 (8)	0.0427 (4)	
N2	0.66369 (7)	0.0716 (4)	0.36798 (9)	0.0482 (5)	
N3	0.68329 (6)	0.5915 (3)	0.28798 (8)	0.0385 (4)	
N4	0.58234 (6)	0.5174 (4)	0.23053 (8)	0.0418 (4)	
N5	0.46527 (7)	0.6261 (5)	0.12918 (10)	0.0679 (6)	
N6	0.46809 (7)	1.2638 (5)	0.01592 (10)	0.0657 (6)	
C1	0.74396 (18)	0.1886 (11)	0.4101 (2)	0.0444 (5)	0.516 (4)
C2	0.7750 (3)	0.2928 (12)	0.4002 (2)	0.0565 (14)	0.516 (4)
H2	0.7624	0.3876	0.3605	0.068*	0.516 (4)
C3	0.8246 (2)	0.2582 (13)	0.4482 (3)	0.0568 (13)	0.516 (4)
H3	0.8458	0.3294	0.4414	0.068*	0.516 (4)
C4	0.84309 (16)	0.1195 (12)	0.5063 (3)	0.0611 (7)	0.516 (4)
H4	0.8770	0.0959	0.5391	0.073*	0.516 (4)
C5	0.81201 (19)	0.0153 (11)	0.5163 (2)	0.0596 (12)	0.516 (4)
H5	0.8246	−0.0795	0.5559	0.072*	0.516 (4)
C6	0.76244 (18)	0.0498 (11)	0.4682 (2)	0.0551 (11)	0.516 (4)
H6	0.7412	−0.0214	0.4750	0.066*	0.516 (4)
C1A	0.74487 (19)	0.1788 (12)	0.4091 (2)	0.0444 (5)	0.484 (4)
C2A	0.7774 (3)	0.3742 (10)	0.4168 (3)	0.0565 (14)	0.484 (4)
H2A	0.7660	0.5350	0.3884	0.068*	0.484 (4)
C3A	0.8267 (3)	0.3344 (11)	0.4661 (4)	0.0568 (13)	0.484 (4)
H3A	0.8490	0.4679	0.4714	0.068*	0.484 (4)
C4A	0.84344 (17)	0.0991 (13)	0.5077 (3)	0.0611 (7)	0.484 (4)
H4A	0.8771	0.0719	0.5414	0.073*	0.484 (4)
C5A	0.8109 (2)	−0.0963 (11)	0.5000 (2)	0.0596 (12)	0.484 (4)
H5A	0.8223	−0.2571	0.5284	0.072*	0.484 (4)
C6A	0.76159 (19)	−0.0564 (11)	0.4507 (3)	0.0551 (11)	0.484 (4)
H6A	0.7393	−0.1900	0.4454	0.066*	0.484 (4)
C7	0.66759 (7)	0.4096 (4)	0.31061 (9)	0.0385 (5)	
C8	0.61906 (7)	0.3724 (4)	0.28314 (10)	0.0395 (5)	
C9	0.61930 (8)	0.1579 (4)	0.32169 (11)	0.0458 (5)	
C10	0.57752 (8)	0.0445 (5)	0.31473 (12)	0.0575 (6)	
H10A	0.5884	−0.1118	0.3461	0.086*	
H10B	0.5530	−0.0286	0.2703	0.086*	
H10C	0.5636	0.1991	0.3232	0.086*	
C11	0.64643 (7)	0.7328 (4)	0.23600 (9)	0.0360 (4)	
C12	0.64814 (7)	0.9456 (4)	0.19683 (9)	0.0368 (4)	
C13	0.68659 (7)	1.0487 (4)	0.20208 (10)	0.0424 (5)	
H13	0.7185	0.9834	0.2359	0.051*	
C14	0.67732 (8)	1.2503 (5)	0.15671 (10)	0.0470 (5)	
H14	0.7032	1.3228	0.1594	0.056*	
C15	0.63093 (8)	1.3466 (5)	0.10772 (10)	0.0480 (5)	
H15	0.6255	1.4850	0.0774	0.058*	
C16	0.59220 (7)	1.2445 (4)	0.10213 (10)	0.0440 (5)	
H16	0.5604	1.3125	0.0684	0.053*	
C17	0.60064 (7)	1.0419 (4)	0.14650 (9)	0.0387 (5)	
C18	0.56731 (7)	0.8908 (4)	0.15189 (9)	0.0389 (5)	
C19	0.59741 (7)	0.6983 (4)	0.20825 (9)	0.0379 (4)	
C20	0.51870 (7)	0.9165 (4)	0.11397 (10)	0.0421 (5)	
C21	0.49030 (8)	0.7526 (5)	0.12444 (11)	0.0499 (5)	
C22	0.49111 (8)	1.1104 (5)	0.05965 (11)	0.0491 (5)	

### Atomic displacement parameters (Å^2^)

**Table:**

	*U*^11^	*U*^22^	*U*^33^	*U*^12^	*U*^13^	*U*^23^
N1	0.0487 (10)	0.0408 (9)	0.0452 (10)	0.0038 (8)	0.0331 (9)	0.0037 (8)
N2	0.0606 (12)	0.0437 (10)	0.0563 (11)	0.0011 (9)	0.0447 (11)	0.0022 (9)
N3	0.0421 (9)	0.0351 (9)	0.0406 (9)	0.0017 (7)	0.0274 (8)	−0.0007 (7)
N4	0.0452 (10)	0.0383 (9)	0.0471 (10)	−0.0017 (8)	0.0318 (9)	−0.0063 (8)
N5	0.0494 (12)	0.0848 (16)	0.0745 (15)	−0.0056 (11)	0.0418 (12)	−0.0071 (12)
N6	0.0538 (12)	0.0703 (14)	0.0579 (13)	0.0127 (11)	0.0287 (11)	0.0066 (12)
C1	0.0527 (13)	0.0398 (12)	0.0459 (12)	0.0081 (10)	0.0339 (11)	0.0016 (9)
C2	0.0545 (17)	0.058 (3)	0.060 (3)	0.018 (2)	0.037 (2)	0.020 (3)
C3	0.0543 (17)	0.058 (3)	0.063 (4)	0.015 (2)	0.040 (2)	0.006 (2)
C4	0.0583 (15)	0.0649 (17)	0.0510 (14)	0.0200 (13)	0.0306 (13)	0.0071 (13)
C5	0.073 (2)	0.056 (3)	0.047 (3)	0.017 (3)	0.038 (2)	0.008 (2)
C6	0.0618 (18)	0.054 (3)	0.050 (3)	0.006 (2)	0.036 (2)	0.006 (2)
C1A	0.0527 (13)	0.0398 (12)	0.0459 (12)	0.0081 (10)	0.0339 (11)	0.0016 (9)
C2A	0.0545 (17)	0.058 (3)	0.060 (3)	0.018 (2)	0.037 (2)	0.020 (3)
C3A	0.0543 (17)	0.058 (3)	0.063 (4)	0.015 (2)	0.040 (2)	0.006 (2)
C4A	0.0583 (15)	0.0649 (17)	0.0510 (14)	0.0200 (13)	0.0306 (13)	0.0071 (13)
C5A	0.073 (2)	0.056 (3)	0.047 (3)	0.017 (3)	0.038 (2)	0.008 (2)
C6A	0.0618 (18)	0.054 (3)	0.050 (3)	0.006 (2)	0.036 (2)	0.006 (2)
C7	0.0458 (11)	0.0339 (10)	0.0412 (11)	0.0034 (9)	0.0302 (10)	−0.0016 (9)
C8	0.0460 (12)	0.0344 (10)	0.0469 (12)	0.0009 (9)	0.0337 (10)	−0.0038 (9)
C9	0.0551 (13)	0.0421 (11)	0.0521 (13)	−0.0012 (10)	0.0398 (12)	−0.0045 (10)
C10	0.0674 (15)	0.0569 (14)	0.0712 (16)	−0.0045 (12)	0.0548 (14)	−0.0006 (12)
C11	0.0381 (11)	0.0328 (10)	0.0377 (10)	0.0001 (8)	0.0245 (9)	−0.0044 (8)
C12	0.0396 (11)	0.0326 (10)	0.0381 (10)	−0.0005 (8)	0.0247 (9)	−0.0043 (8)
C13	0.0393 (11)	0.0429 (11)	0.0410 (11)	−0.0017 (9)	0.0238 (10)	−0.0040 (9)
C14	0.0468 (12)	0.0497 (12)	0.0511 (13)	−0.0024 (10)	0.0342 (11)	−0.0006 (10)
C15	0.0541 (13)	0.0479 (12)	0.0446 (12)	0.0024 (10)	0.0327 (11)	0.0035 (10)
C16	0.0442 (12)	0.0431 (11)	0.0404 (11)	0.0038 (10)	0.0249 (10)	−0.0008 (9)
C17	0.0392 (11)	0.0352 (10)	0.0407 (11)	0.0004 (8)	0.0250 (9)	−0.0051 (9)
C18	0.0410 (11)	0.0352 (10)	0.0409 (11)	−0.0011 (8)	0.0262 (10)	−0.0088 (9)
C19	0.0394 (11)	0.0345 (10)	0.0409 (11)	0.0003 (8)	0.0261 (10)	−0.0042 (8)
C20	0.0391 (11)	0.0420 (11)	0.0411 (11)	0.0023 (9)	0.0238 (10)	−0.0052 (9)
C21	0.0392 (12)	0.0558 (13)	0.0516 (13)	−0.0009 (11)	0.0274 (11)	−0.0101 (11)
C22	0.0412 (12)	0.0528 (13)	0.0481 (13)	0.0026 (11)	0.0261 (11)	−0.0072 (11)

### Geometric parameters (Å, º)

**Table:**

N1—C7	1.361 (2)	C4A—C5A	1.3900
N1—N2	1.391 (2)	C4A—H4A	0.9500
N1—C1	1.403 (5)	C5A—C6A	1.3900
N1—C1A	1.430 (5)	C5A—H5A	0.9500
N2—C9	1.311 (3)	C6A—H6A	0.9500
N3—C11	1.328 (2)	C7—C8	1.409 (3)
N3—C7	1.353 (2)	C8—C9	1.426 (3)
N4—C19	1.327 (2)	C9—C10	1.489 (3)
N4—C8	1.342 (3)	C10—H10A	0.9800
N5—C21	1.147 (3)	C10—H10B	0.9800
N6—C22	1.148 (3)	C10—H10C	0.9800
C1—C2	1.3900	C11—C19	1.421 (3)
C1—C6	1.3900	C11—C12	1.466 (3)
C2—C3	1.3900	C12—C13	1.384 (3)
C2—H2	0.9500	C12—C17	1.413 (3)
C3—C4	1.3900	C13—C14	1.390 (3)
C3—H3	0.9500	C13—H13	0.9500
C4—C5	1.3900	C14—C15	1.382 (3)
C4—H4	0.9500	C14—H14	0.9500
C5—C6	1.3900	C15—C16	1.387 (3)
C5—H5	0.9500	C15—H15	0.9500
C6—H6	0.9500	C16—C17	1.384 (3)
C1A—C2A	1.3900	C16—H16	0.9500
C1A—C6A	1.3900	C17—C18	1.474 (3)
C2A—C3A	1.3900	C18—C20	1.356 (3)
C2A—H2A	0.9500	C18—C19	1.466 (3)
C3A—C4A	1.3900	C20—C22	1.434 (3)
C3A—H3A	0.9500	C20—C21	1.437 (3)
			
C7—N1—N2	110.14 (16)	N1—C7—C8	106.37 (17)
C7—N1—C1	131.2 (3)	N4—C8—C7	123.33 (18)
N2—N1—C1	118.4 (3)	N4—C8—C9	130.67 (19)
C7—N1—C1A	131.0 (3)	C7—C8—C9	105.99 (18)
N2—N1—C1A	118.8 (3)	N2—C9—C8	109.54 (18)
C9—N2—N1	107.95 (16)	N2—C9—C10	122.5 (2)
C11—N3—C7	110.46 (16)	C8—C9—C10	128.0 (2)
C19—N4—C8	111.93 (16)	C9—C10—H10A	109.5
C2—C1—C6	120.0	C9—C10—H10B	109.5
C2—C1—N1	120.3 (4)	H10A—C10—H10B	109.5
C6—C1—N1	119.7 (4)	C9—C10—H10C	109.5
C3—C2—C1	120.0	H10A—C10—H10C	109.5
C3—C2—H2	120.0	H10B—C10—H10C	109.5
C1—C2—H2	120.0	N3—C11—C19	124.72 (17)
C2—C3—C4	120.0	N3—C11—C12	127.38 (17)
C2—C3—H3	120.0	C19—C11—C12	107.90 (16)
C4—C3—H3	120.0	C13—C12—C17	120.84 (18)
C5—C4—C3	120.0	C13—C12—C11	130.85 (18)
C5—C4—H4	120.0	C17—C12—C11	108.30 (16)
C3—C4—H4	120.0	C12—C13—C14	118.35 (19)
C6—C5—C4	120.0	C12—C13—H13	120.8
C6—C5—H5	120.0	C14—C13—H13	120.8
C4—C5—H5	120.0	C15—C14—C13	120.9 (2)
C5—C6—C1	120.0	C15—C14—H14	119.5
C5—C6—H6	120.0	C13—C14—H14	119.5
C1—C6—H6	120.0	C14—C15—C16	121.1 (2)
C2A—C1A—C6A	120.0	C14—C15—H15	119.4
C2A—C1A—N1	121.3 (4)	C16—C15—H15	119.4
C6A—C1A—N1	118.5 (4)	C17—C16—C15	118.82 (19)
C1A—C2A—C3A	120.0	C17—C16—H16	120.6
C1A—C2A—H2A	120.0	C15—C16—H16	120.6
C3A—C2A—H2A	120.0	C16—C17—C12	119.95 (18)
C4A—C3A—C2A	120.0	C16—C17—C18	131.13 (18)
C4A—C3A—H3A	120.0	C12—C17—C18	108.91 (17)
C2A—C3A—H3A	120.0	C20—C18—C19	125.14 (18)
C5A—C4A—C3A	120.0	C20—C18—C17	128.95 (19)
C5A—C4A—H4A	120.0	C19—C18—C17	105.91 (16)
C3A—C4A—H4A	120.0	N4—C19—C11	124.33 (18)
C4A—C5A—C6A	120.0	N4—C19—C18	126.69 (17)
C4A—C5A—H5A	120.0	C11—C19—C18	108.98 (17)
C6A—C5A—H5A	120.0	C18—C20—C22	122.7 (2)
C5A—C6A—C1A	120.0	C18—C20—C21	123.22 (19)
C5A—C6A—H6A	120.0	C22—C20—C21	114.06 (18)
C1A—C6A—H6A	120.0	N5—C21—C20	176.0 (2)
N3—C7—N1	128.40 (18)	N6—C22—C20	178.4 (2)
N3—C7—C8	125.22 (18)		
			
C7—N1—N2—C9	−0.6 (2)	N3—C7—C8—C9	178.93 (17)
C1—N1—N2—C9	174.4 (2)	N1—C7—C8—C9	0.0 (2)
C1A—N1—N2—C9	177.4 (3)	N1—N2—C9—C8	0.6 (2)
C7—N1—C1—C2	−18.7 (4)	N1—N2—C9—C10	−177.98 (18)
N2—N1—C1—C2	167.6 (2)	N4—C8—C9—N2	178.80 (19)
C1A—N1—C1—C2	69 (11)	C7—C8—C9—N2	−0.3 (2)
C7—N1—C1—C6	160.6 (3)	N4—C8—C9—C10	−2.8 (4)
N2—N1—C1—C6	−13.1 (4)	C7—C8—C9—C10	178.1 (2)
C1A—N1—C1—C6	−112 (11)	C7—N3—C11—C19	0.2 (3)
C6—C1—C2—C3	0.0	C7—N3—C11—C12	−179.09 (17)
N1—C1—C2—C3	179.3 (4)	N3—C11—C12—C13	1.4 (3)
C1—C2—C3—C4	0.0	C19—C11—C12—C13	−177.97 (19)
C2—C3—C4—C5	0.0	N3—C11—C12—C17	−179.69 (18)
C3—C4—C5—C6	0.0	C19—C11—C12—C17	0.9 (2)
C4—C5—C6—C1	0.0	C17—C12—C13—C14	0.2 (3)
C2—C1—C6—C5	0.0	C11—C12—C13—C14	178.95 (19)
N1—C1—C6—C5	−179.3 (4)	C12—C13—C14—C15	0.3 (3)
C7—N1—C1A—C2A	14.8 (4)	C13—C14—C15—C16	−0.3 (3)
N2—N1—C1A—C2A	−162.6 (3)	C14—C15—C16—C17	−0.3 (3)
C1—N1—C1A—C2A	−80 (11)	C15—C16—C17—C12	0.8 (3)
C7—N1—C1A—C6A	−169.9 (3)	C15—C16—C17—C18	−178.22 (19)
N2—N1—C1A—C6A	12.7 (4)	C13—C12—C17—C16	−0.8 (3)
C1—N1—C1A—C6A	95 (11)	C11—C12—C17—C16	−179.76 (17)
C6A—C1A—C2A—C3A	0.0	C13—C12—C17—C18	178.45 (17)
N1—C1A—C2A—C3A	175.2 (4)	C11—C12—C17—C18	−0.6 (2)
C1A—C2A—C3A—C4A	0.0	C16—C17—C18—C20	−0.9 (3)
C2A—C3A—C4A—C5A	0.0	C12—C17—C18—C20	179.99 (19)
C3A—C4A—C5A—C6A	0.0	C16—C17—C18—C19	179.1 (2)
C4A—C5A—C6A—C1A	0.0	C12—C17—C18—C19	0.0 (2)
C2A—C1A—C6A—C5A	0.0	C8—N4—C19—C11	0.8 (3)
N1—C1A—C6A—C5A	−175.3 (4)	C8—N4—C19—C18	−179.82 (17)
C11—N3—C7—N1	179.02 (18)	N3—C11—C19—N4	−0.8 (3)
C11—N3—C7—C8	0.3 (3)	C12—C11—C19—N4	178.58 (17)
N2—N1—C7—N3	−178.56 (18)	N3—C11—C19—C18	179.68 (17)
C1—N1—C7—N3	7.3 (4)	C12—C11—C19—C18	−0.9 (2)
C1A—N1—C7—N3	3.8 (4)	C20—C18—C19—N4	1.1 (3)
N2—N1—C7—C8	0.4 (2)	C17—C18—C19—N4	−178.91 (18)
C1—N1—C7—C8	−173.8 (3)	C20—C18—C19—C11	−179.43 (18)
C1A—N1—C7—C8	−177.2 (3)	C17—C18—C19—C11	0.6 (2)
C19—N4—C8—C7	−0.3 (3)	C19—C18—C20—C22	179.49 (18)
C19—N4—C8—C9	−179.28 (19)	C17—C18—C20—C22	−0.5 (3)
N3—C7—C8—N4	−0.3 (3)	C19—C18—C20—C21	−0.6 (3)
N1—C7—C8—N4	−179.24 (17)	C17—C18—C20—C21	179.43 (18)

### Hydrogen-bond geometry (Å, º)

**Table:**

*D*—H···*A*	*D*—H	H···*A*	*D*···*A*	*D*—H···*A*
C10—H10*A*···N5^i^	0.98	2.69	3.362 (3)	126

Symmetry code: (i) −*x*+1, *y*−1, −*z*+1/2.
