# Supplementary figures and images for: Crystal structure of 2-[12-methyl-14-phenyl-10,13,14,16-tetra­aza­tetra­cyclo[7.7.0.02,7.011,15]hexa­deca-1(16),2,4,6,9,11(15),12-heptaen-8-yl­idene]propandi­nitrile
Source: Acta Crystallogr Sect E Struct Rep Online. 2014 Nov 8;70(Pt 12):o1244–5. doi: 10.1107/S1600536814024167 (PMC4257392; doi:10.1107/S1600536814024167)

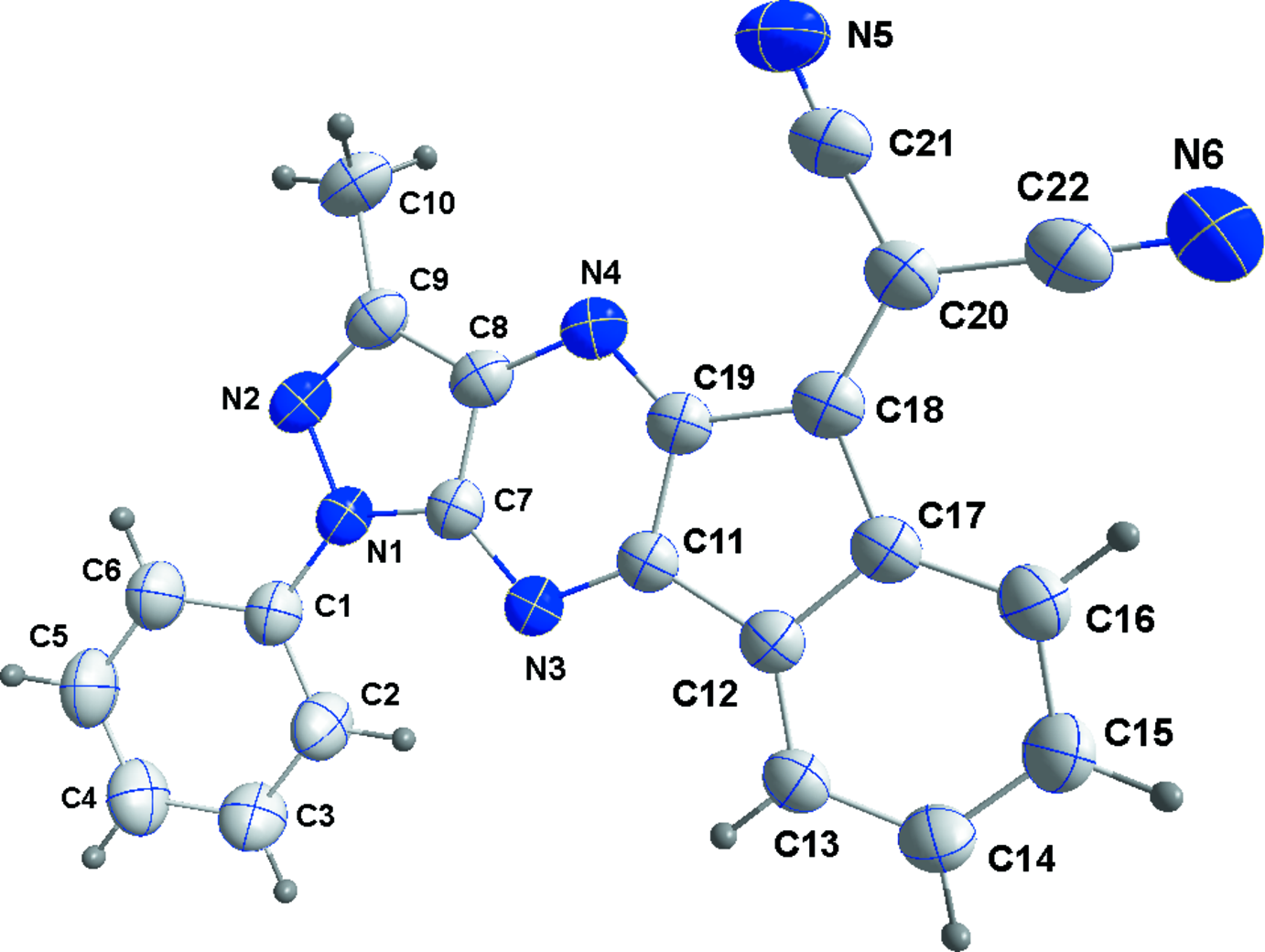

Supplement: Supplementary file 4 [file e-70-o1244-fig1.tif]

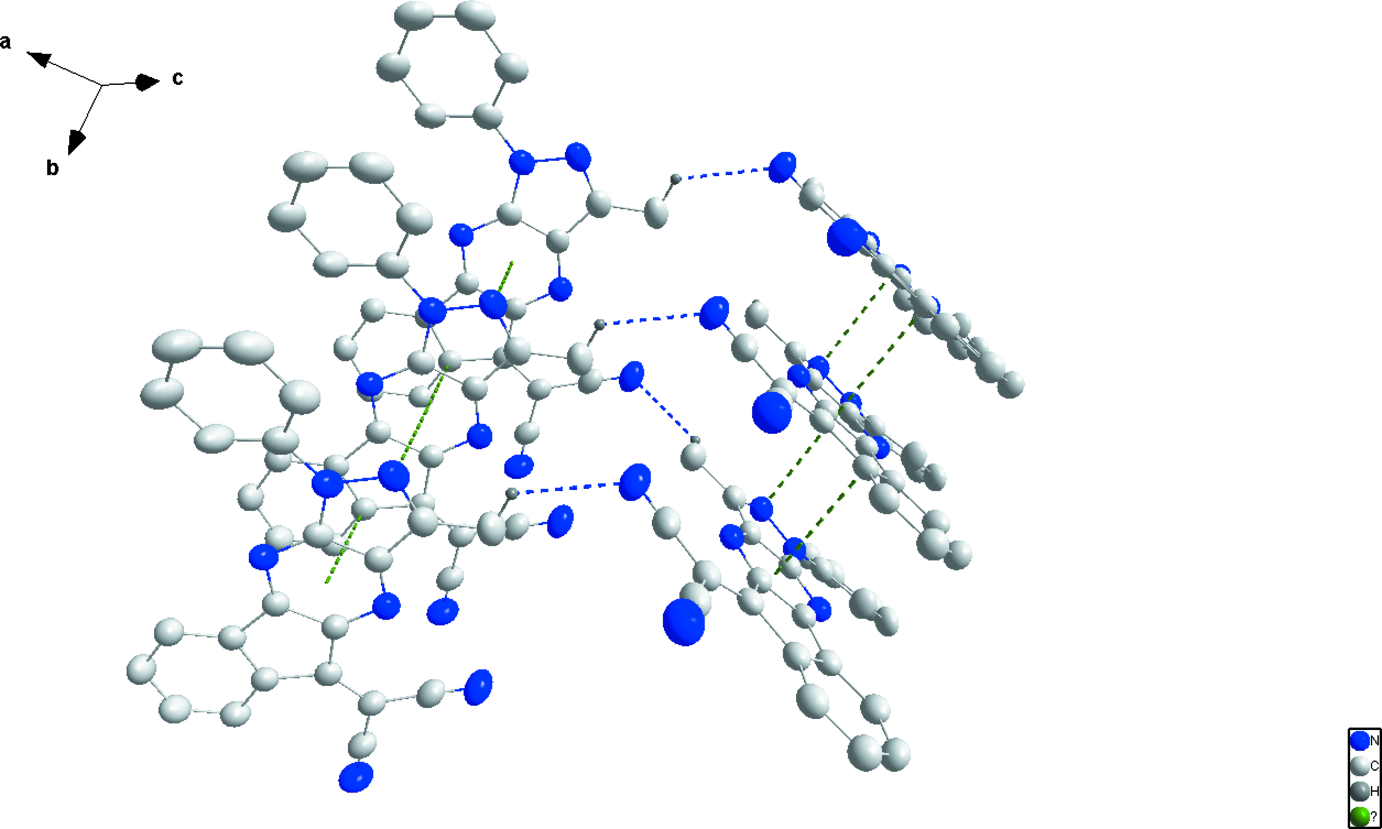

Supplement: Supplementary file 5 [file e-70-o1244-fig2.tif]

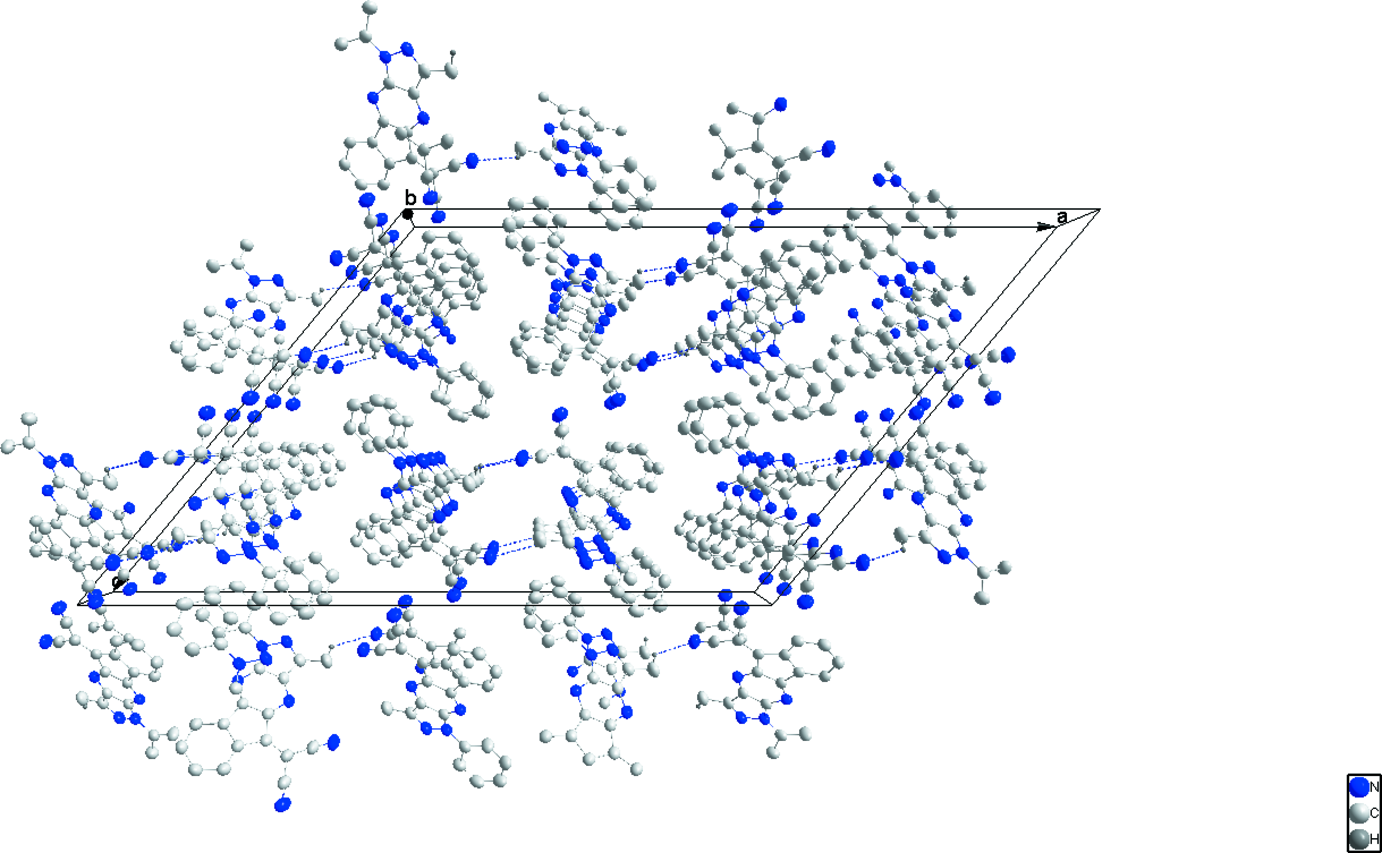

Supplement: Supplementary file 6 [file e-70-o1244-fig3.tif]
